# A rare and novel mutation in a beta-globin gene of thalassemia patient of Pakistan: A case report

**DOI:** 10.1016/j.amsu.2022.104918

**Published:** 2022-11-17

**Authors:** Arsala Rashid, Shehroze Tabassum, Aroma Naeem, Asif Naveed, Haris Iqbal, Shehram Tabassum, Humera Rafiq

**Affiliations:** a King Edward Medical University, Pakistan; b University of Health Sciences, Pakistan; c Faisalabad Medical University, Pakistan; d Punjab Institute of Neurosciences, Pakistan

**Keywords:** Thalassemia, HBB, c.-138C>A, Thalassemia intermedia, Rare mutation, Case report

## Abstract

**Introduction:**

Thalassemia is a genetically complex disorder that evolves from a mutation in the beta chain of hemoglobin. Much work has been done on the common mutations, but some rare mutations have been found that impact and diversify the disease spectrum.

**Case presentation:**

Our case report is on a young adult who presented with anemia, gall stones, and off-and-on transfusion dependency. A detailed workup revealed that the patient was suffering from thalassemia intermedia. The interesting finding was that the patient, product of non-consanguineous marriage was homozygous for beta thalassemia mutation on genetic analysis. A detailed genetic analysis of the parents revealed them as carriers for the same mutation. It was found that patient was homozygous for a rare and novel mutation −88(C > A)[HBB:c.-138C  >  A] on whole gene sequencing.

**Discussion:**

The area of genomics in thalassemia is rapidly growing, and our case report aims to update the current knowledge of thalassemia's genomic information in Pakistan. The mutation found in our patient was −88(C > A)[HBB:c.-138C  >  A], and the data provided by the National Library of Medicine for this mutation as Allele ID: 380597 and variant type of single nucleotide variant shows that only ten such cases exist in the world with this rare mutation. Our case would be the 11th case in the world and 1st in Pakistan according to the literature, reporting above mentioned mutation.

**Conclusion:**

Further translational study is required to accurately utilize genomic data as an instrument of precision treatment in thalassemia patients, especially in underdeveloped countries like Pakistan.

## Highlights

• Thalassemia evolves from a mutation in the beta chain of hemoglobin transferred mostly from parents to children or rarely a de novo mutation might occur.
• Globally there are more than 535 gene mutations in beta chain of hemoglobin with common ones in Pakistan being IVSI-1,IVSI-5, IVSII-1, del 619, Fr 8–9, Fr 16, Fr 41–42.
• Our case report is about a thalassemia patient having a rare mutation in beta globin gene of hemoglobin on genetic analysis.

## 1 Introduction

Beta thalassemia, an autosomal recessive disorder, occurs as a consequence of genetically defective or reduced production of the beta-globin chains of hemoglobin [1]. It is the most usual single-gene disorder with >400,000 new-cases [2]. Geographical distribution includes malarial belts, equatorial areas of the Mediterranean region, the Middle East, the Trans Caucasus, a few central parts of Asia, the subcontinent, and Southeast Asia [3]. The microarray sequence of a beta gene consists of three exons with two intervening sequences (IVS1 and IVS2) and the 5′ and 3′ untranslated regions. Serious hemoglobinopathies such as sickle cell anemia and β-thalassemia occur due to defects in the β globin chain of the hemoglobin. This gene is located on chromosome 11, whose defect causes abnormal assembly or inadequate production of hemoglobin [2]. Not many years ago, a phenomenological terminology for beta-thalassemia was coined, the presence of two abnormal alleles, B^0^ and B^+.^ The B^0^ thalassemia is regarded as the one which virtually does not produce any protein, and B^+^ thalassemia indicates mutation, consequently producing a decreased quantity of beta globin protein. Additionally, three groups of beta-thalassemia have been identified: beta-thalassemia major, intermedia, and minor. Clinically thalassemia is differentiated by the spectrum of anemia into three classes. On one end, thalassemia major patients have severe anemia; on the other, thalassemia minor patients suffer from mild anemia. Meanwhile, thalassemia intermedia patients are those who lie intermediate in reference to severity between β-thalassemia trait and β-thalassemia major [4]. Thalassemia intermedia and major might have a converging clinical spectrum [5]. Globally there are more than 535 gene mutations in the beta chain of hemoglobin. Within South Asia, there are approximately 45 × 10^6^ carriers of beta thalassemia [6,7]. Such a large number of carriers make a carrier rate of ∼1:20. Frequently, there are four navels for beta-thalassemia mutations in this region: g.63201_63819del619 (619bp deletion), c.27_28insG, c.92+1G > A, and c.92+5G > C. Literature shows a high consanguineous product rate (81%) in cases from the Indian subcontinent and Pakistan [8]. The polymorphism at c.92+5G > C was most frequent (37.0%) for the beta gene, with the second most common one being c.27_28insG and 619base pair omission, thus having allele frequencies of about 20% and 10–15%, respectively. The carrier rate of β-thalassemia in Pakistan is 5%. It differs among racial groups. Balochs have the highest prevalence (8%) [9]. This case report has been written in accordance with SCARE guidelines [10].

### 1.1 Case

A male patient, 22 years old well oriented in time, place and space, resident of Lahore District, presented to us from an outdoor patient department (OPD) with pallor, yellowish discoloration of sclera for the last four years, and recent onset of pain in the right hypochondrium. Blood transfusions have been given every sixth month for the last four years. The patient was referred to the hematology section from surgical OPD for an opinion regarding cholecystectomy and management of persistent anemia with mild splenomegaly. He had history of three to four transfusion in last 5 years. He was a product of non-consanguineous marriage with two elder sisters, which were both normal. His complete blood count (CBC) results are shown in Table 1. The laboratory tests were carried out in a specialized laboratory of the tertiary care hospital in which patient presented. The tests were verified and inter-observer difference was minimized by two independent experts (having an experience of five years) viewing the reports and smears on two different occasions.

**Table 1:** CBC results of the patient.

Blood Parameters	Results
Hemoglobin	5.4 g/dl
Peripheral Blood Smear	Microcytosis++
	Macrocytosis ++
	Anisopoikilocytosis +++
	Target cells +
	Tear drop +
	Reticulocyte count: 35%
	Corrected RC: 15.5%
DLC	Polymorphs: 80%
	Lymphocytes: 09%
	Monocytes: 05%
	Eosinophils: 06%
Serum Iron	78 ng/dl
TIBC	189.0 μg/dl
Serum Ferritin	265 ng/ml
Total Bilirubin	4.6mg/dl
Direct Bilirubin	0.7 mg/dl
Indirect Bilirubin	3.9 mg/dl
LDH	575 U/L

**Fig. 1:**
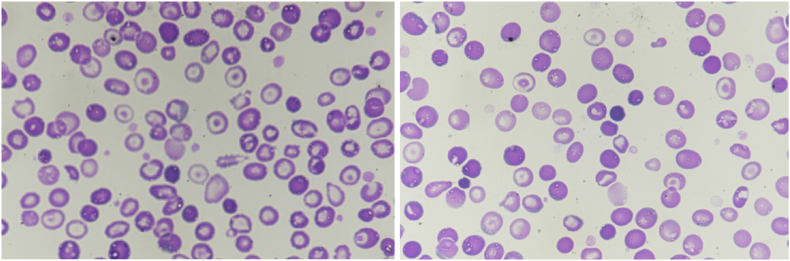
Pictures from Peripheral Blood film stained with Giemsa stain showing a predominant population of hypochromic microcytic red blood cells (RBCs) and prominent target cells.

**Fig. 2:**
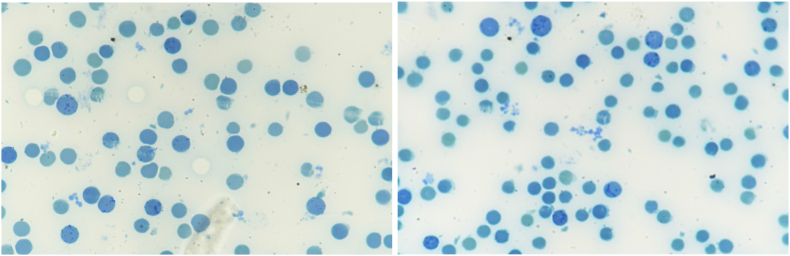
Pictures of supravital stain showing increased reticulocyte count while the patient was on therapy with folic acid.

His peripheral blood and reticulocyte images are shown below: (see Fig. 1) (see Fig. 2)

**Fig. 3:**
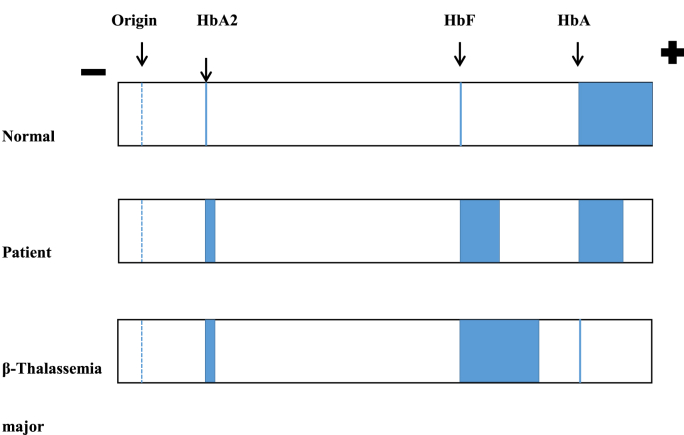
Electrophoresis Pattern of our patient and Comparison with Normal person and β-Thalassemia major patient.

The patient had no history of drug intake other than hematemics, including vitamin B12 and folic acid. He had a history of three to four transfusions in last five years. However, even with the provided treatment, patient had persistent anemia. There was no history of thalassemia in the family. Keeping in mind the presentation of the patient with anemia, high reticulocyte count, and mild splenomegaly, the possibility of autoimmune hemolytic anemia, enzymopathies, membranopathies, and genetic defects of hemoglobin was considered. The Direct and Indirect Coombs Tests were negative. Urine Examination showed no free hemoglobin or RBCs in urine. The absence of spherocytes and osmotic fragility test showed no increased osmotic fragility of RBCs. The patient was tested for Glucose-6-phosphate dehydrogenase (G6PD) assays after the reticulocyte count was back in a normal range and the acute episode was settled. G6PD assays came out to be 17.7 U/gHb and were within the normal range. The electrophoresis showed HBA 48.5%, HBF 47.2%, and HBA2 4.3%.(see Fig. 3)

Analysis for genetic mutations i.e., IVSI-1, IVSI-5, IVSII-1, del 619, Fr 8–9, Fr 16, Fr 41–42, Cd 5, Cd 15, cd 30, Cap+1, IVSI-25, Fr 47–48,-88, cd 39, Hb-S, Hb-C, Hb-E, and Hb-D (Punjab), that are commonly seen in Pakistan was done and none of these mutations were detected in the patient. Analysis of the rest of the family was also done for genetic mutations. The mother and father showed indices like thalassemia trait, with the father showing slightly raised HbA2 on High-performance Liquid Chromatography. Molecular analysis of common Beta-Thalassemia mutations was done for both parents, and they also did not carry any of the common Beta-thalassemia mutations. The patient was tested for alpha thalassemia, too; however, there was no alpha gene deletion or duplication found in the patient.

A whole general sequencing was then carried out. Mutation in this patient was identified by genetic analysis as −88(C > A)[HBB:c.-138C  >  A]. The patient was homozygous for the mutation. The genetic analysis of the parents showed that both parents were carriers of the same mutation −88(C > A)[HBB:c.-138C  >  A]. Thus, the patient being homozygous for the mutation was labeled as thalassemia major. However, clinically he was found to have thalassemia intermedia.

## 2 Discussion

The presentation of the patient was unusual as the parents had a non-consanguineous marriage. Moreover, there was no family history of thalassemia. This case report highlights the importance of thalassemia and its rare mutations prevailing in Pakistan. Variability in clinical presentation and hematological characteristics in Thalassemia Major and Intermedia indicates that such diseases can present with a broad range of disease manifestations. There is much overlap between the two, especially with certain mutations and confounding factors. In Pakistan, six uncommon mutants of a beta gene are Cd 15 (G-A), Cd 30 (G-C), Cd 5 (-CT), Fr 16 (-C), Cap+1 (A-C), and Hb-E [11]. These unusual genetic abnormalities are responsible for 4% of all genes. Cd 30 (G-A), IVSII–I (G-A), IVSI-1 (G-A), and Cd 37 (G-A) being seldom found are the alterations reported so far [12]. The population of Pakistan has a 5% carrier rate for Beta-thalassemia, 8.3% for alpha-thalassemia with only one gene deletion, and 2% for two gene deletion (-α3.7α/-α3.7α) [13]. The presence of HbD is mainly confined to Punjab (1%) [12]. Considering the 5% carrier rate of Beta-thalassemia, it is calculated that 5000 new births of thalassemia major will occur yearly.

In our patient, the mutation found was −88(C > A)[HBB:c.-138C  >  A], and the data provided by the National Library of Medicine for this mutation as Allele ID: 380597 and variant type of single nucleotide variant shows that only ten such cases exist in the world with this rare mutation [14]. Rund et al. first documented this rare mutation in a study conducted on the genetic development of beta-thalassemia in the Jews of Kurdistan in 1991 in the world [15]. It is one of the rarest mutations detected. This rare mutation has never been detected in Pakistan, and to the best of our knowledge, it will be the 11th case going to be reported in the world so far [14]. In a study by Moatter, a rare mutation on the same locus of −88(C-T) [HBB: c.-138C  >  G] was reported in prenatal screening for β-thalassemia major patients [16]. Among the different thalassemia mutations, a C-T transition in the same base has been discovered and reported in Bahrain, Egypt, and Iran [17]. The mutation, i.e., C- - - A transversion in an upstream promoter element at −88 (CACACCC), is a slight change in DNA which results in a less severe ß+ characteristic (Thalassemia intermedia). Thus, in our case, the patient being homozygous for the mutation and being thalassemia major, had a different and variable presentation than the classical presentation. Both parents were carriers for the mutations revealed by the genetic analysis. Since none of the other siblings of the patient were affected, it is a huge possibility that, unfortunately, the mutation might have arisen in both or single parents spontaneously. Such cases highlight the need and importance of detailed genetic analysis, especially in underdeveloped and thalassemia prevalent countries. Due to a lack of diagnostic facilities, rare and less severe mutations may go undiagnosed, eventually increasing the disease burden in society. It remains an essential tool in identifying all such mutations which may confound the disease spectrum and its severity.

## 3 Conclusion

Our report identified infrequent and novel mutation that will be useful in finding cases of thalassemia in Pakistan and will help to find out the exact prevalence of the disease. This rare mutation is first one in Pakistan and maybe an eye opener to look for the mutation in thalassemia patients with unusual presentations. The case report also emphasizes to focus on the prevalence and proper detection of cases of thalassemia intermedia so that its appropriate treatment may also be carried out. Although presentations of thalassemia intermedia are usually less severe than thalassemia major, they can present with many complications later in life. So, an early detection by looking into possible signs of anemia and analyzing the genetics will help the hematologists to develop proper strategy regarding managing such rare cases that have low frequency in specific regions of the world. Moreover, this study will also help in adding to the existing knowledge of geographical distribution of such rare mutations of beta-globin gene, finding associated risk factors of such mutations and window-period of complications that appear years after birth.

## Ethical approval

Not required as we have acquired consent from the patient.

## Source of funding

None.

## Author contributions

Writing: Arsala Rashid, Shehroze Tabassum, Aroma Naeem, Asif Naveed, Haris Iqbal, Shehram Tabassum, Humera Rafiq. Review with Critical Comments: Arsala Rashid, Shehroze Tabassum. Editing: Arsala Rashid, Shehroze Tabassum.

## Research registration

1. Name of the registry: N/A
2. Unique Identifying number or registration ID: N/A
3. Hyperlink to your specific registration (must be publicly accessible and will be checked): N/A

## Guarantor

Shehroze Tabassum.

## Consent

Written informed consent was obtained from the patient for publication of this case report and accompanying images. A copy of the written consent is available for review by the Editor-in-Chief of this journal on request.

## Provenance and peer review

Not commissioned, externally peer reviewed.

## Declaration of competing interest

None.
